# Exploring the reasons for defaulting from childhood immunization: a qualitative study in Pakistan

**DOI:** 10.1186/s12889-024-17926-y

**Published:** 2024-02-08

**Authors:** Kifayat Ullah, Javeria Saleem, Rubeena Zakar, Muhammad Ishaq, Farhad Ali Khattak, Fatima Majeed, Hafiza Aisha Sadiqa, Florian Fischer

**Affiliations:** 1 Department of Public Health, Institute of Social and Cultural Studies, University of the Punjab, Lahore, Pakistan; 2 Institute of Social and Cultural Studies, University of the Punjab, Lahore, Pakistan; 3 Khyber College of Dentistry, Peshawar, Pakistan; 4 Institute of Public Health, Charité – Universitätsmedizin Berlin, Charitéplatz 1, Berlin, 10117 Germany

**Keywords:** Defaulter, Vaccination, Immunization, Vaccine hesitancy, Expanded program on immunization

## Abstract

**Background:**

Childhood vaccination is widely recognized as the most effective means to prevent various diseases. However, a considerable amount of children still miss out on their vaccination schedules. Therefore, this study explores the reasons for defaulting from the expanded program on immunization in district Swat, Pakistan.

**Methods:**

A qualitative phenomenological approach was used. Data collection took place from March to September 2022. Thirty-six in-depth interviews were conducted with participants who had defaulter children. The collected qualitative data were analysed thematically to identify key patterns and themes related to the reasons for defaulting from childhood vaccination schedules.

**Results:**

Six themes emerged, i.e., illness of the defaulter child at the scheduled time, perceived side effects of the vaccination, factors related to caregivers, myths and misconceptions, vaccinators attitudes and crowed vaccination centres, as well as poor immunization service arrangements. Four subthemes arose related to caregivers, such as lack of clear understanding about completion of vaccination, least priority for child’s vaccination, cultural restriction on mothers, and the loss of vaccination card.

**Conclusion:**

According to the study’s findings, caregivers have their own perceptions regarding the non-completion of their children’s vaccination schedule. The childhood immunization defaulting arises from various factors including child illness, Adverse Events Following Immunization (AEFIs) concerns, misconceptions, improper injection techniques, and negative vaccinator attitudes. The vaccination completion rate may be increased if the concerns of the caregivers are appropriately addressed.

## Background

The health status of individuals, particularly children, significantly depends on vaccination against vaccine preventable diseases (VPDs). Data suggests that VPDs are one of the significant contributing factors to mortality and morbidity in countries with low vaccination coverage [1]. Between 2010 and 2018, approximately 18 million children died of VPDs, despite the availability of cost-effective vaccination that could prevent these outcomes [2]. Like, the successful eradication of smallpox and the limited prevalence of poliomyelitis in a few countries are notable achievements of vaccination [3]. Immunization serves as a crucial preventive measure against early childhood disease. Therefore, vaccination programs have played a pivotal role in averting millions of child deaths and disabilities worldwide [4].

To mitigate childhood diseases, the World Health Organization (WHO) initiated the Expanded Program on Immunization (EPI) in 1974 and Pakistan started EPI program in 1978. Despite being a cost-effective health strategy that improves survival rates, many children in developing countries are unable to receive the complete series of recommended vaccines and become defaulter [5]. The phrase “defaulter child” is used operationally by vaccination programs to refer to a child who does not receive the entire vaccination series [6]. A significant number of children remained defaulters despite initiating vaccination and receiving early doses [7]. Data reveal that 12.4 million children who have not received the full combination vaccines against the three infectious diseases diphtheria, pertussis, and tetanus (DPT-3) are from ten countries: Nigeria, India, Pakistan, Indonesia, Ethiopia, Congo, Angola, Iraq, South Africa, and Afghanistan. Notably, 7% of these unimmunized children live in Pakistan [8].

According to a study conducted in Nepal, the coverage for the first dose of diphtheria, pertussis, and tetanus (DPT-1) stands at 98%, whereas the coverage for the third dose (DPT-3) drops to 83%, indicating a significant dropout rate between these two doses [9]. In Pakistan, pneumonia, diarrhoea, and meningitis cause more than half of all fatalities in post neonatal infants, which can be prevented by vaccination [10]. Data from Pakistan reveal that only 66% of children aged 12–23 months receive complete immunization [11], marking the lowest rate among the countries in the subcontinent [12]. The national Bacillus Calmette-Guérin (BCG) vaccine coverage is 88%, while measles coverage is 73%. Furthermore, the coverage of vaccines has been adversely impacted by the COVID-19 pandemic, leading to additional crises and increased morbidity and mortality [13].

According to data from 2018, the estimated global dropout rate for DPT vaccines was 4%. This means that more than five million infants initiated the vaccination but did not complete the recommended schedule [14]. Sub-Saharan Africa, Latin America, and the Caribbean exhibited 8% DPT dropout. Among the 5.9 million children who did not complete the entire series of DPT vaccines, more than 70% resided in Africa and South Asia [15]. The dropout rate varies significantly among countries. Low- and middle-income countries have the highest DPT dropout rates. A study conducted in Malaysia reported defaulter rates ranging from 16.8% to 24.8%, with a consistent increase in defaulters from BCG to the third dose, i.e., 4.8%, 10.5%, and 22.4%, respectively [16], while in Ethiopia, the first- to third-dose dropout rate was 43% [17].

There are multiple factors contributing to low vaccination rates among children. Parental knowledge, attitude, and socioeconomic status have been linked with low vaccination rates among children [5]. Staff absences, a shortage of clinic personnel, and insufficient duty hours also resulted in missed opportunities to vaccinate children [18]. Program policies such as visits by appointment only, waiting for a time appointed to them, specific days for vaccination, the limit on per day vaccination, long waiting hours for vaccination, and vaccinations administered only by trained technicians, have an adverse impact on vaccine coverage and utilization [19]. Negative views regarding the health care system and less belief in susceptibility to various diseases by parents also affect vaccination coverage inversely [20].

It is crucial to determine the reasons why caregivers do not complete the vaccination schedule for their children. Therefore, this study aimed to determine the reasons for defaulting from childhood immunization in the Swat district, Pakistan.

## Methods

### Study design

A qualitative phenomenological approach was used within this study. Interviews were conducted from March to September 2022. The phenomenological approach was chosen because it allows for the exploration of subjective experiences and perspectives of the participants, enabling a first-person version of the data [21]. To explore the reasons for defaulting, we conducted 36 in-depth interviews with parents who had defaulter children. The interviews were discontinued once data saturation was reached, as some participants began reiterating information already expressed in previous interviews. Information regarding defaulter children was obtained from the vaccination centres of the selected union councils, and visits were made to their homes for interviews.

### Study area and population

This study was conducted in the Swat district, which has the third largest population in the province of Khyber Pakhtunkhwa, Pakistan. District Swat, being a neglected and marginalized region in Khyber Pakhtunkhwa, faces unique challenges due to its rugged terrain, hilly areas, and freezing weather, making it difficult for vaccinators to conduct outreach sessions and for the community to visit the health facility for vaccination. The district was previously hit by terrorism and militancy, which severely affected the functioning of the health system. The vaccinators and Lady Health Workers (LHWs) were warned to be killed if they conducted field visits for vaccination [22].

The study population includes caregivers with children under the age of two who had not completed their children’s vaccination. This group was chosen to gather first-hand information because these caregivers initiate early immunization but fail to complete the recommended vaccination schedule. Thus, it is believed that they may have specific reasons that led them to discontinue vaccinating of their children [23]. Participants who expressed unwillingness to participate in the study and those who had migrated from other districts were excluded from the study.

### Sampling technique

A cluster sampling technique was adopted to select the Union Councils (UCs). The UCs in the district were initially categorized into two groups: urban and rural. From each group, two UCs were randomly selected using a simple random sampling method. Subsequently, vaccination centers within the chosen UCs were visited, and defaulter children were identified by referring to the EPI and LHW registers. Pertinent information such as the child’s name, father’s name, age, gender, address, and vaccination status was recorded, and a list of defaulter children was compiled.

### Data collection procedure and tools

For the in-depth interviews, a semi-structured interview guide was employed. The development of the interview guide involved an extensive review of literature on which the formulation of research questions and objectives was based upon. The interview guide was designed in English and later translated into Urdu. Prior to commencing the actual data collection, pilot interviews were conducted. Based on the feedback from the pilot interviews, minor adjustments were made to the interview guide, including alterations to the sequence of questions. Participants were informed about the interviews through local LHWs before visiting their homes for the interviews. Verbal consent was obtained from the participants prior to the interview. The study’s purpose and the data’s confidentiality were explained to the participants. Participants were assured that they could withdraw from the study at any time without providing a reason. Two of the participants refused the interview without explaining the reasons. The interviews were conducted by the first author of this contribution and were recorded using a tape recorder. Field notes were also taken during the interviews. The average duration of the interviews ranged from 18 to 31 min. Subsequently, the principal investigator transcribed the interviews.

### Data analysis

For data analysis, a thematic analysis approach was utilized, and the software NVivo-12 was employed. Thematic analysis is a flexible and valuable qualitative research approach [24]. The goal of thematic analysis was to make sense of the interviews and to identify themes related to the reasons for not completing the children’s vaccination schedule. All 36 recorded interviews along with field notes were transcribed and used for analysis. To become familiar with the data, the researchers engaged in repeated listening and revising of the data. Initial codes were identified during this process. These codes were discussed and validated among the authors, subsequently merged, and categorization was performed. Multiple categories were then combined by the authors to identify the final themes. To avoid missing data, the generated themes were reviewed and matched with the primary data again. The final themes were defined and given names that accurately conveyed their meaning. The names assigned to the themes were chosen to be easily understandable and self-explanatory while maintaining the essence of the underlying concepts. Excerpts and interview quotes were included to provide further explanation and support for the findings.

## Results

The average age of the defaulter children was 13.5 months, ranging from 3 to 24 months, while the average age of the participants was 42.7 years, ranging from 21 to 75 years. Of the 36 interview participants, 26 (72.2%) were male, while 10 (27.8%) were female. The interviews were conducted with caregivers, which were either father (*n* = 12, 33.3%), grandfather (*n* = 9, 25.0%), mother (*n* = 6, 16.7%), uncle (*n* = 6, 16.7%), or grandmother (*n* = 3, 8.3%). Of the 36 families, 86.1% (*n* = 31) were joint families, while 13.9% (*n* = 5) were nuclear families (Table 1).

**Table 1:** Demographic table of the interview’s participants

Interview code	Age in years	Gender	Relation with defaulter child	Family type (Joint/Nuclear)	Urban/Rural
PR-01	35	Male	Father	Joint	Urban
PR-02	55	Male	Grandfather	Joint	Urban
PR-03	43	Male	Uncle	Joint	Urban
PR-04	60	Female	Grandmother	Joint	Urban
PR-05	41	Male	Father	Joint	Urban
PR-06	34	Male	Father	Nuclear	Urban
PR-07	65	Male	Grandfather	Joint	Urban
PR-08	30	Female	Mother	Joint	Urban
PR-09	35	Female	Mother	Nuclear	Urban
PR-10	21	Male	Uncle	Joint	Urban
PR-11	30	Male	Father	Joint	Urban
PR-12	59	Male	Grandfather	Joint	Urban
PR-13	31	Male	Father	Nuclear	Urban
PR-14	70	Male	Grandfather	Joint	Urban
PR-15	35	Male	Uncle	Joint	Urban
PR-16	55	Female	Grandmother	Nuclear	Urban
PR-17	75	Male	Grandfather	Joint	Urban
PR-18	26	Male	Uncle	Joint	Urban
PR-19	24	Male	Father	Joint	Rural
PR-20	65	Female	Grandmother	Joint	Rural
PR-21	52	Male	Grandfather	Joint	Rural
PR-22	37	Male	Father	Joint	Rural
PR-23	22	Female	Uncle	Joint	Rural
PR-24	35	Female	Mother	Joint	Rural
PR-25	36	Male	Uncle	Joint	Rural
PR-26	36	Male	Father	Joint	Rural
PR-27	30	Male	Father	Joint	Rural
PR-28	30	Male	Father	Joint	Rural
PR-29	38	Female	Mother	Joint	Rural
PR-30	45	Female	Father	Nuclear	Rural
PR-31	37	Male	Father	Joint	Rural
PR-32	70	Male	Grandfather	Joint	Rural
PR-33	60	Male	Grandfather	Joint	Rural
PR-34	35	Female	Mother	Joint	Rural
PR-35	21	Male	Uncle	Joint	Rural
PR-36	62	Male	Grandfather	Joint	Rural

Using all 36 in-depth interviews, six main themes and four subthemes as reasons for defaulting from childhood immunization were categorized (Table 2), which are described in detail in the following part. The labels provided in Table 2 refer to the number of participant, age, and sex (e.g., “PR-04, 60, F” indicates that participant number 4 is female and has an age of 60 years).

**Table 2:** Themes identified by merging codes and categories

Themes	Codes and categories merged as a theme	Number of participants contributing (*n* = 36)	Interview code
Illness of the defaulter child at the scheduled time	The child was ill at the scheduled vaccination time	13	PR-01, PR-06, PR-12, PR-13, PR-14, PR-17, PR-21, PR-23, PR-24, PR-26, PR-30, PR-33, PR-35
Perceived side effects of the vaccination	Side effects following initial doses of the vaccines	11	PR-03, PR-04, PR-07, PR-20, PR-22, PR-26, PR-29, PR-31, PR-32, PR-33, PR-36
Factors related to parents	Carelessness by the caregiver, Unawareness of the caregivers regarding the vaccination status of the child, Unavailability of the father of the child, Timely vaccination of the child is not the priority of the caregiver, Caregivers are busy with other domestic tasks, Difficulty in visiting the vaccination center, Misplaced vaccination card, The vaccination card was not checked for the scheduled date by the caregiver	24	PR-02, PR-03, PR-04, PR-05, PR-06, PR-08, PR-09, PR-10, PR-11, PR-14, PR-15, PR-17, PR-18, PR-19, PR-20, PR-22, PR-23, PR-24, PR-25, PR-27, PR-31, PR-32, PR-34, PR-36
Myths and misconceptions regarding vaccination	Myths and misconceptions of the caregivers	6	PR-05, PR-12, PR-21, PR-22, PR-25, PR-26
Attitude of vaccinators and crowed vaccination centers	Rude attitude of the vaccinators, Crowded vaccination centers,	4	PR-15, PR-16, PR-22, PR-27
Poor immunization services arrangements	Poor services arrangements at the vaccination center, Late outreach sessions in the area, Unavailability of the child at UC	4	PR-22, PR-28, PR-33, PR-35

### Illness of the defaulter child at the scheduled time

One of the predominant reasons for deviating from the routine vaccination schedule was the occurrence of various illnesses in children during the scheduled vaccination time. Out of the 36 participants, 13 shared that their child was unwell, leading them to postpone or skip the vaccine. Many participants expressed uncertainty regarding the potential benefits or risks of vaccinating a sick child, ultimately opting not to administer the vaccine during the illness.

The main illnesses reported during the study included gastroenteritis, chest infections, high-grade temperature, acute hepatitis, and whooping cough. Two children had cardiac diseases, and one child had undergone surgery for large bowel obstruction. It is worth noting that most of these illnesses are not considered contraindications for the vaccines included in EPI. It was found that vaccines were also not given to twin children because the parents thought that the children were malnourished and very weak:

“*If my child has high-grade temperature, how can I allow giving a vaccine dose to him? If I do, the child will die.*” (PR-06, 34, M)

The participants expressed concerns about their child’s health and believed that administering vaccines during illness might exacerbate the child’s discomfort. Many participants lacked awareness of the contraindications related to vaccinations. They did not consult their doctors or health care providers about vaccinating the child during the illness. However, two participants mentioned consulting their doctors about their child’s vaccination, and they were advised not to administer the vaccines during the illness. One of the children was malnourished, and the other had cardiac disease. When asked if they had consulted any healthcare provider regarding their child’s vaccination, the participant with a child suffering from a cardiac disease (septal defect) responded:

“*The vaccinator told me not to vaccinate your child during the disease as it may adversely affect your child*.” (PR-23, 22, M)

The caregivers were unaware, and neither the vaccinators nor health care providers counselled them in which conditions the vaccines are contraindicated and may be delayed.

### Perceived side effects of the vaccination

One of the themes identified was the perceived side effects of the vaccination, as most of the participants were worried about Adverse Events Following Immunization (AEFI). Participants were asked whether their child or any other child they knew had experienced any side effects following vaccination. Thirteen out of the 36 participants responded affirmatively. The most commonly reported AEFI was fever, which was mentioned by ten participants:

“*The child was suffering from fever and was crying for two days when the previous dose of vaccine was given.*” (PR-07, 65, M)

Participants believed that if they proceeded with the vaccination, there was a possibility that their child might experience a high-grade fever, leading to a delay of the vaccination:

“*We had a wedding ceremony in the family at the scheduled vaccination time, and if we give vaccines, the child might have developed high-grade fever and we would not be able to enjoy the wedding ceremony; therefore, we postponed the vaccination at the scheduled time.*” (PR-04, 60, F)

One participant shared a specific experience in which their child developed an abscess at the site of the BCG vaccine and experienced a high-grade fever. Subsequently, the child was taken to a paediatrician who admitted the child for two days. The child received intravenous injections and underwent minor surgery to clean the wound at the injection site.

### Factors related to parents

The incomplete vaccination status of the child was attributed to parental factors, which emerged as a significant theme. Among the majority of participants (23 out of 36), incomplete vaccination was acknowledged as a result of parental neglect rather than deliberate refusal. The interviews revealed four subthemes here.

#### Lacking a clear understanding of the completion of vaccination

Four interviews highlighted the theme of parents lacking a clear understanding of complete vaccination for their children. These interviews revealed that these parents were already convinced of the benefits of vaccination and considered it crucial for their child’s well-being. They expressed their willingness to adhere to the vaccination schedule and complete it on time. Although these parents held a positive attitude towards childhood vaccination, they admitted that they had not practiced timely completion of their child’s EPI vaccination. When asked why their child’s vaccination was incomplete, they attributed it to their own negligence.

“*We have never thought about the completion of our child’s vaccination, but I cannot explain why?*” (PR-15, 35, M)

Despite being aware of the significance of vaccination and its timely completion, the parents’ carelessness resulted in their child not receiving a complete schedule of vaccinations.

#### Least priority for child’s vaccination

Three participants expressed that they were unable to take their child to the vaccination center due to other competing priorities, such as:

“*I am a mason and go to work early in the morning and come back in the evening; how I can manage time for taking the child to the hospital for vaccination?* […] *Only on Friday I have off but having so many tasks to do in a single off day.*” (PR-11, 30, M).

Another participant stated:

“*The mother of the child is diabetic and unable to take the child for vaccination, and I [father] am too busy; therefore, the child defaulted from the program.*” (PR-19, 24, M)

The participants’ responses indicated that child vaccination ranked low in their list of priorities compared to other tasks. For example, one of the participants who worked six days a week and had one day off did not prioritize their child’s vaccination. Despite having a weekly day off, they managed their time for other family-related tasks but neglected to ensure their child’s vaccination.

#### Cultural restrictions on mothers

The defaulting from the vaccination program in the Swat district was also attributed to cultural restrictions. Four participants highlighted that mothers of children were required to be accompanied by a male family member when visiting vaccination centers, as per cultural norms. This restriction posed a barrier to mothers accessing vaccination services independently and contributed to their inability to comply with the vaccination program.

“*Males are mostly busy, and females alone are not allowed to take the child to the vaccination centre.*” (PR-17, 75, M)

Some participants revealed that the absence of the child’s father, who was out of the city, coupled with cultural norms, prevented mothers from attending the vaccination center. These cultural norms restricted women from leaving their homes without their husbands, leading to their inability to access vaccination services independently.

“*I [father of the child] was in Karachi at the time of my child’s vaccination, and my wife was not allowed to go to the vaccination centre without me. Therefore, the time of vaccination was missed. In addition, I know vaccination of the child has its importance but what we can do as our females cannot go outside without male?*” (PR-31, 37, M)

The requirement for females to be accompanied by a male family member had an adverse impact on completing the vaccination schedule. This cultural norm contributes to the limited utilization of vaccination services, ultimately leading to a high number of defaulter children.

#### Loss of vaccination card

The vaccination card serves as the sole official document for parents to maintain a record of their children’s vaccination. The entries on the card are made by the vaccinator during the vaccination process. However, twelve participants identified various issues related to the vaccination card:

“*The vaccination card of the child was misplaced in the home, and we were not aware that either the child would get vaccinated without the card or not.*” (PR-02, 55, M)

The vaccination card plays a crucial role as the sole record containing vaccination dates. However, if the card is lost or becomes unreadable, parents may face challenges in determining the child’s next vaccination date. While they can visit the vaccination center to confirm the date, this process requires additional effort for both caregivers and vaccinators. The vaccinator needs to search the child’s record in the daily and permanent EPI registers at the vaccination center to provide the necessary information.

Nine participants reported forgetting the vaccination dates and not checking the vaccination card for the next visit:

“*The child was vaccinated at the vaccination centre, and when I returned home, I checked the card, the date for the next dose was not written. Therefore, I was unaware of when to visit the vaccination centre for the next dose.*” (PR-25, 36, M)

When asked why he had not revisited the vaccination center to confirm the date of the next dose, the response was as follows:

“*I thought that the vaccination schedule may be completed and no further vaccines will be given to the child; therefore, the vaccinator did not mention the date for the next visit.*” (PR-25, 36, M)

Incomplete documentation confuses caregivers and parents about the vaccination schedule, leading to children missing doses.

Two participants mentioned their children’s vaccination cards being with the local LHW, leaving them unaware of the next vaccination date. Proper documentation and communication are vital to avoid such issues and ensure timely vaccinations.

### Myths and misconceptions regarding vaccination

Six out of thirty participants expressed reservations about the vaccination program. Despite receiving the initial doses of vaccines for their children, they were uncertain about continuing the vaccination process due to misconceptions and doubts:

“*The foreign countries support this program, and if a foreign country is financially supporting to vaccinate our children, they must have some hidden interests. For these hidden interests, they are donating money to vaccinate our children.*” (PR-21, 52, M)

The participants were unsure about the undisclosed motives foreign countries might have in children’s vaccination. However, they remained hesitant to place complete trust in the vaccination program.

The constituents of the vaccines were identified as a factor contributing to children defaulting from the vaccination program:

“*No one knows about the constituents of vaccines and nor can it be tested at any laboratory, so how we can trust it.”* (PR-26, 36, M)

“*Previously, there was no vaccination, and we have never been vaccinated and are healthy and spending our life normally. Therefore, there is no need for any vaccination. Like us, our children will also spend their life without vaccination.*” (PR-12, 59, M)

Two participants expressed concerns that vaccines could lead to early puberty and sterility in children. They believed that vaccinating their children might result in such issues occurring later in life. These perceived adverse effects of vaccination, particularly in terms of early puberty and sterility in male children, have generated worries within the community. As a result, some parents choose not to pursue further immunization for their children.

### Attitude of vaccinators and crowed vaccination centres

Defaulting from vaccination was attributed to the behavior of the vaccinator and inappropriate injection techniques. The defaulter rate increased due to the vaccinator’s rude behaviour and inappropriate injection technique. People avoided visiting vaccination centers because of these negative experiences with the vaccinator:

“*They are giving injections like giving to the animals, and the child cries very badly; it should not be given like a sting. I was distraught when the injection was given to my child.*” (PR-15, 35, M)

Long waiting times and overcrowded vaccination centers were cited as factors contributing to child defaulting from the immunization program. Three participants reported enduring lengthy waits for their children’s turn to be vaccinated, potentially leading to parents avoiding vaccination visits. The participant expressed concern about the lack of adequate waiting areas and seating arrangements for clients at the vaccination center. The overcrowded and limited space in the waiting area discourages parents from returning to the center for their child’s next vaccination dose:

“*It was challenging to manage time to visit the vaccination centre for my child’s vaccination, but at the centre, I had to wait for hours. It is not easy to wait for a long time along with the child.*” (PR-22, 37, M)

### Poor immunization services arrangements

A well-structured service delivery mechanism is a crucial factor for improving vaccination services. Three participants reported that their children were not at home on the scheduled vaccination dates. When asked why they did not vaccinate their child in the adjacent UC, two of them were unaware that vaccination could be done in another UC. Meanwhile, one participant visited the nearby UC vaccination center, but the vaccinator refused to vaccinate the child:

“*We visited the nearby health facility to get the child vaccinated in the adjacent UC. The vaccinator told us that your child can only be vaccinated in the UC where the initial vaccines were given to the child, and the child has been registered in that UC.*” (PR-28, 30, M)

The lack of proper and timely conduction of outreach sessions was a factor contributing to defaulting from the immunization program. Community members reported waiting for the next outreach session but were unaware of the date for the upcoming session:

“*Previously, the child was vaccinated in an outreach session, but now no outreach session was conducted in our area. The previous session conducted here was almost six months back.*” (PR-22, 37, M)

## Discussion

This study aimed to investigate the factors behind incomplete childhood vaccination schedules. Six main themes were identified which were related to the child (illness at the scheduled time and [perceived] side effects), related to the parents (i.e. lacking a clear understanding of the completion of vaccination, least priority for child’s vaccination, cultural restrictions on mothers, and loss the vaccination card), related to social aspects and norms (i.e. myths and misconceptions about immunization programs), and finally relation to vaccination centres and services themselves (i.e. attitudes of vaccinators, crowding at vaccination centres, and poor immunization services arrangements). However, most of these dimensions are also intertwingled.

The most common cause of defaulting from routine immunization was the child’s illness at the time of vaccination, primarily due to infectious diseases. Participants were unaware of the effects of vaccines when administered during illnesses. Many of the diseases mentioned could have allowed vaccination with a slight delay. Participants might have vaccinated their children if they had received proper counselling from vaccinators or health care providers. A similar study conducted in Southern Ethiopia also reported that mothers lacked counselling and practical information about vaccines and the suitable conditions for their administration [21]. According to our study, malnourished children were not given vaccines, but proper counselling could have led to timely vaccination. Health workers must address parents’ concerns and provide information about vaccine contraindications to reduce missed vaccination opportunities. In our study, some participants received advice from physicians or vaccinators to delay vaccination, but the exact timing was not explained, causing confusion about when to vaccinate the child after recovery from illness. Another study in Nigeria also reported that 3.6% of children dropped out of the vaccination program due to ill health, supporting our findings and providing a specific percentage of children who defaulted due to health issues [25].

The study found that adverse events following immunization (AEFI), such as fever and abscess at the injection site, contributed to dropping out from the vaccination program. Poor counselling by the vaccinator regarding these adverse effects resulted in participants leaving the program. Similar findings were reported in a study which emphasized that injection neuritis could be prevented through proper injection techniques and vaccinators adopting aseptic injection practice [26].

This study identified several parental factors leading to non-completion of child vaccination at the scheduled time. These factors included parental carelessness and prioritizing other tasks over vaccination, as well as cultural sensitivity regarding female empowerment. During the study, participants acknowledged the importance of completing vaccination but failed to do so for their children, indicating parental carelessness regarding vaccination. Some participants mentioned having many other tasks and being unable to manage time for their child’s vaccination, suggesting that vaccination was given low priority. Similar findings were reported by a study conducted in Ghana, where 14.4% of children were not vaccinated due to parents being occupied with other tasks [27]. Cultural restrictions prohibiting females from going outside the home without a male partner were identified as a reason for dropping out from vaccination. Similar findings were reported in a study which highlighted the impact of cultural sensitivity that prevents females from being in a position to vaccinate their children. These restrictions adversely affect child vaccination rates [28].

The proper care of vaccination cards by parents or vaccinators significantly impacts vaccine coverage. Parents who miss the vaccination card are often unaware if the vaccination can proceed without it. A study conducted in Ethiopia highlighted that missing the EPI card is a major cause of dropping out from the vaccination program. Some parents believe the card is essential, and vaccinating the child may be refused if they do not have it. Parents who lose the card may feel hesitant to visit the vaccination center due to fear and potential rude behavior from vaccinators. The study also concluded that the issue of missing vaccination cards has been neglected in Ethiopia, and the vaccination program lacks a proper policy or mechanism for vaccinating children who have lost their cards [21]. In Pakistan’s vaccination program, similar to Ethiopia, there is a lack of appropriate tools and clear policies for vaccinating children who have lost their vaccination cards. Proper entry into the vaccination card by the vaccinator is essential for completing the vaccine schedule. The study revealed that missing entries by the vaccinator regarding the date for the next dose contributed to dropping out from the vaccination program. Parents were unaware of when to administer the next dose of vaccines, even though they believed their child’s vaccination had been completed. The vaccinator’s failure to mention the date for the next visit led to this confusion and dropout.

Myths and misconceptions related to childhood immunization pose significant challenges to vaccination programs, especially in low-income countries. In this study, participants expressed concerns about the constituents of vaccines. While they had given their children initial doses of EPI vaccines, they discontinued further vaccination due to doubts created by aid from donor countries and international non-governmental organizations. They believed that foreign aid must come with hidden interests, even though they could not explain the nature of those interests. Lack of awareness about vaccine constituents also negatively impacted community trust in vaccinations. Some participants believed vaccines could cause sterility or early puberty in the future. Trust plays a crucial role in influencing parents’ attitudes towards childhood immunization. Most people in Pakistan think that vaccination causes sterilization in children as it has been described in a study which quantified the number of defaulters due to trust deficits in vaccination programs [29].

Poor crowd management and long waiting times at vaccination centers discourage parents from making timely visits. Waiting for a long time along with children in overcrowded centres is challenging for parents and leads to dissatisfaction [30]. In our study, participants perceived vaccination centers as consistently crowded, with vaccinators administering vaccines in a disorganized manner. The absence of a proper mechanism for managing the clients’ turn results in everyone trying to get their child vaccinated first, leading to chaos and dissatisfaction. Furthermore, the attitude and behaviour of the vaccinator and healthcare provider significantly impact parents’ confidence and attachment to the immunization program. A previous study reported that 14.3% of children dropped out of the program due to disrespectful behavior from vaccinators [31]. Similarly, in our study, we found that vaccinators’ unfriendly attitude towards parents of children resulted in dropping out from the program.

Poor immunization services arrangements emerged as a factor contributing to dropping out from the vaccination program. In this study, it was observed that vaccinators refused to vaccinate children if they had received initial doses in another vaccination center. This missed opportunity occurred when the child visited the center but was not vaccinated because they belonged to the catchment area of another center. Late and unplanned outreach sessions were also identified as factors increasing the dropout rate. These sessions likely caused inconvenience for parents, leading to missed vaccination opportunities.

### Strengths and limitations

The study was conducted in nonclinical settings, specifically in participants’ households, to ensure a comfortable and pressure-free environment during the interviews. This helped to create a conducive and respectful atmosphere for the participants, facilitating open and honest responses during the interviews. Additionally, the sample was quite large for a qualitative study. Nevertheless, it is recognized that there are some limitations of the study as well. The results may be specific to Khyber Pakhtunkhwa and may not be directly generalizable to other provinces in the country due to significant differences in socioeconomic status and cultural factors. However, these limitations do not impact the validity of the current study’s findings within the context of Khyber Pakhtunkhwa.

The results might be biased in a way that we were only able to recruit those people wo where willing to talk about childhood immunization.

## Conclusion

Addressing the reasons behind defaulting is crucial to improve vaccination rates. Raising awareness about the diseases for which vaccines can be given, along with proper counselling about AEFI and vaccination cards, can help reduce the number of defaulters. Developing mechanisms for vaccination and establishing data-sharing mechanisms with parents’ can facilitate efficient vaccination. Furthermore, promoting polite behavior and safe injection techniques among vaccinators may encourage parents to bring their children for the next vaccination, fostering confidence in the immunization program. These strategies combined can contribute to increasing vaccination coverage and improving overall public health outcomes.

## Data Availability

Data is available from corresponding author upon reasonable request.

## Abbreviations

AEFI: Adverse Events Following Immunization
BCG: Bacillus Calmette-Guérin
DPT: Diphtheria, pertussis, and tetanus
EPI: Extended Program on Immunization
LHW: Lady Health Worker
UC: Union Council
VPD: Vaccine preventable disease
WHO: World Health Organization
